# Associations of physical performance and physical activity with mental well-being in middle-aged women

**DOI:** 10.1186/s12889-021-11485-2

**Published:** 2021-07-23

**Authors:** Dmitriy Bondarev, Sarianna Sipilä, Taija Finni, Urho M. Kujala, Pauliina Aukee, Vuokko Kovanen, Eija K. Laakkonen, Katja Kokko

**Affiliations:** 1 Gerontology Research Center and Faculty of Sport and Health Sciences, University of Jyväskylä, P.O. Box 35 (viv 152), FI-40014 Jyväskylä, Finland; 2 Neuromuscular Research Center, Faculty of Sport and Health Sciences, University of Jyvaskyla, P.O. Box 35 (viv), FI-40014 Jyväskylä, Finland; 3 Faculty of Sport and Health Sciences, University of Jyväskylä, P.O. Box 35 (viv), FI-40014 Jyväskylä, Finland; 4 Department of Obstetrics and Gynecology, Pelvic Floor Research and Therapy Unit, Central Finland Central Hospital, Hoitajantie 3, FI-40620 Jyväskylä, Finland

**Keywords:** Depressive symptoms, Life satisfaction, Negative affectivity, Positive affectivity, Physical performance, Middle-age women, Aerobic capacity

## Abstract

**Background:**

To investigate whether physical performance is independently of physical activity (PA) associated with positive and negative dimensions of mental well-being in middle-aged women.

**Methods:**

Data were drawn from the Estrogenic Regulation of Muscle Apoptosis (ERMA) study in which women 47 to 55 years were randomly selected from the Finnish National Registry. They (*n* = 909) participated in measurements of physical performance (handgrip force, knee extension force, vertical jumping height, maximal walking speed, and six-minute walking distance). Both mental well-being (the Centre for Epidemiologic Studies Depression Scale, the International Positive and Negative Affect Schedule Short Form and the Satisfaction with Life Scale) and PA were self-reported. Associations between variables were analysed using multivariate linear regression modelling adjusted for body height, fat mass %, menopausal status and symptoms, marital status, parity, employment status, self-reported mental disorders, and use of psycholeptics and psychoanaleptics. PA was then entered into a separate model to explore its role in the associations.

**Results:**

In the adjusted models, significant positive associations of six-minute walking distance with positive affectivity (*B* = 0.12, *p* = 0.002) and life satisfaction (*B* = 0.15, *p* = 0.033) were observed. No significant associations were observed between physical performance and depressive symptoms or negative affectivity. PA was positively associated with positive affectivity and life satisfaction and negatively with depressive symptoms across all the physical performance variables.

**Conclusions:**

Of the physical performance dimensions, aerobic component was associated with positive mental well-being independently of PA level. In relation to other physical performance components, the results point to the benefits of physical activity for mental well-being.

## Background

Mental well-being is a multidimensional construct with positive dimensions, such as general satisfaction with life and positive affectivity, and with negative dimensions such as mental distress and negative affectivity [1]. Higher mental well-being is associated with better health outcomes (e.g., fewer incidents of coronary heart diseases) and longer life expectancy [2, 3]. Although mental well-being has been shown to be relatively stable across the adult years [4], midlife is considered a life transition point at which the cumulative influence of several factors, including menopausal-related hormonal changes or personal life events, may influence mental well-being [5, 6].

Age-associated decline in physical functioning that results in mobility limitations or disabilities among older people [7] may also be related to low mental well-being [8]. It has been shown that women who transitioned through perimenopause to postmenopause had a decline in muscle strength and muscle power on average by 2–3%, which may suggest that the decline in physical functioning accelerates already during midlife [9, 10]. However, the physical performance of more physically active middle-aged women is greater than that of less physically active peers [11]. Since physical activity in middle-aged women is beneficially associated with both positive mental well-being [12, 13] and greater physical performance, mental well-being may also be higher in better physically performing middle-aged women.

Physical performance and physical activity have a close functional relationship. Despite considerable individual differences in the response to regular physical activity, in general, the higher the intensity and amount of physical activity, the better the physical performance [14]. However, the possible influence of physical performance and physical activity on mental well-being may stem from different sources. Physical activity may be linked to well-being through neurobiological (e.g., release of opioids), psychological (e.g., sense of mastery or emotions) or behavioural mechanisms (e.g., health-related behaviour) [15, 16]. Physical performance, in turn, acts as a mechanism in the relationship between physical activity and well-being [17] and may partially be a product of genetics or the amount of physical activity or other life-style choices. A meta-analysis of intervention studies on the link between physical activity and mental well-being showed that, at advanced ages, an improvement in physical performance induced by exercise interventions is related to improved well-being [18]. However, it has not been fully verified whether the level of physical performance plays a role in mental well-being. As middle adulthood in women coincides with a spontaneous reduction in physical activity that may be related to menopause-associated hormonal deficiency [19] and key personal life events [5], it would be useful to further investigate the independent role of physical performance in mental well-being for middle-aged women.

Thus, the aim of this study was to investigate whether physical performance – independent of physical activity – is associated with positive and negative dimensions of mental well-being, utilising a comprehensive set of physical performance measurements.

## Methods

### Study design and participants

**Fig. 1:**
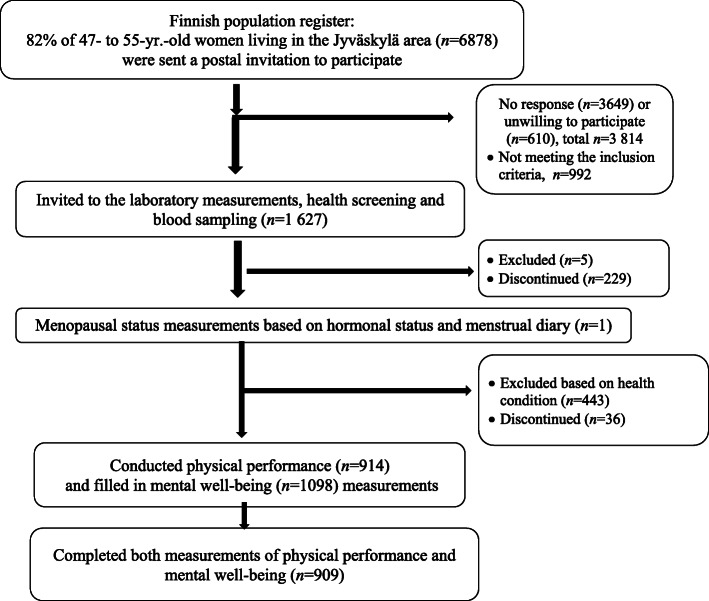
Enrolment of study participants

This study is a part of the Estrogenic Regulation of Muscle Apoptosis (ERMA) study. The ERMA study is a population-based cohort study which consists of the data from women aged 47 to 55 years living in the city of Jyväskylä or neighbouring municipalities in Finland. A detailed description of participant recruitment has been reported earlier [20]. Briefly, an invitation to participate in the study was sent to 6878 women randomly selected from the Finnish National Registry (Fig. 1). Exclusion criteria were a self-reported body mass index (BMI) greater than 35 kg/m^2^, pregnant women or lactating, medical conditions affecting ovarian function (e.g., bilateral ovariectomy, estrogen-containing hormonal preparations), or chronic diseases or medications that may significantly affect muscle function. Among the included participants, 2% reported use of psycholeptics and 7% use of psychoanaleptics medication. Their health status was followed by study physician and they were allowed to participate in the study.

Eligible participants (*n* = 1627) were assessed fasting blood samples in our laboratory and filled in the health screen questionnaire (*n* = 1393). Participants who had reported serious or unclear health conditions were examined by a physician to ensure safe participation in the physical performance measurements. The present analysis concerns women who performed at least one of the physical performance measurements acceptably and completed at least one mental well-being measurement (*n* = 909).

All participants gave their written informed consent. The study protocol followed good clinical and scientific practice and the Declaration of Helsinki and was approved by the Ethics Committee of the Central Finland Health Care District (K-SSHP Dnro 8 U/2014).

### Physical performance

A detailed description of the measurement of physical performance – handgrip and knee extension force, low body muscle power, maximum walking speed and aerobic capacity – has been given earlier [11]. Briefly, *handgrip* and *maximal isometric knee extension forces* were measured in Newtons. Handgrip force was measured on the side of the dominant hand with the participant seated in an adjustable dynamometer chair (Good Strength, Metitur, Palokka, Finland) with elbow flexed at 90°. The participants were instructed to squeeze the handle for 2–3 s to produce maximum force. Knee extension force was measured with the same dynamometer chair with a knee angle set at 60° from full extension. Participants were encouraged to extend the knee to produce maximal force.

*Lower body muscle power* was assessed by a vertical jump on a contact mat. Vertical jumping height was calculated in cm from flight time (*t*): (g × *t*^2^) ÷ 8 × 100.

In all the above tests, three to five maximal attempts were performed and the best performance was taken as the result.

*Maximum walking speed* was measured over 10 m with the instruction to walk “as fast as possible” and timed with photocells. The fastest time of two trials, with self-selected rest of 30–60 s between trials, was taken as the result.

*Aerobic capacity* was assessed with the help of the six-minute walking test. The test was performed on a 20-m indoor track, and participants were encouraged to complete as many laps as they could within 6 min. The distance covered in meters was used in the analysis.

### Mental well-being

*Depressive symptoms* were measured with the 20-item Center for Epidemiological Studies Depression Scale (CES-D [21]). Participants were asked to indicate how often they experienced each symptom over the past week. Each item was scored from 0 = seldom or never to 3 = almost all the time; a mean score for the 20 items was computed. Lower scores corresponded to lower depressive symptomatology.

The International Positive and Negative Affect Schedule Short Form (I-PANAS-SF [22]) was used to measure *positive and negative affectivity.* This inventory comprises five positive affect adjectives and five negative affect adjectives. Participants rated each of these adjectives on a scale from 1 = does not describe me to 5 = describes me very well. Average scores ranging from 1 to 5 were computed for each affective subscale, with lower scores indicating a lower degree to experience positive or negative affectivity.

*Life satisfaction* was measured with the 5-item Satisfaction with Life Scale which intended to evaluate global cognitive judgments of one’s life satisfaction [23]. Participants indicated the degree to which they agree or disagree with each of the 5 items on a scale ranging from 1 = strongly disagree to 7 = strongly agree. The mean score of the scale for the five items was computed, with lower scores corresponding to lower satisfaction with life.

### Physical activity

Level of leisure physical activity was measured on a seven-point scale assessing physical activity patterns from household duties, leisure-time physical activity to competitive sports. The PA questionnaire has been described previously and has been validated in middle-aged women [24]. Briefly, the response categories were: 1 = inactive, 2 = light activity 1 to 2 times per week, 3 = light activity several times per week, 4 = moderate activity 1 to 2 times per week, 5 = moderate activity several times per week, 6 = high activity several times per week, and 7 = competitive sports and related training several times per week. The seven response categories were recoded to form three levels of PA: *low* (categories 1 and 2), medium (3 and 4), and *high* (5 and 7), that were used as a variable for the analysis.

#### Background and adjusted variables

*Age* was calculated from date of birth to the date of the first laboratory visit. *Level of education* was measured with a single question and responces were grouped as primary (primary school), secondary (secondary school) and tertiary (applied science or bachelor’s degree, nurse training, master’s degree or PhD).

*Body height* was measured in meters by a stadiometer. *Body mass* was measured in kilograms with a digital scale with the participant wearing undergarments only.

*Body fat* was measured in percentage of body weight with a multifrequency bioelectrical impedance analyser (InBodyTM 720; Biospace, Seoul, Korea).

*Menopausal status* was measured based on monthly self-reported bleeding patterns and serum follicle stimulating hormone (FSH) concentrations according to the Stages of Reproductive Aging Workshop (STRAW) recommendations [25]. FSH measurements were conducted from fasting serum samples which were collected between 8:00 and 10:00 AM. For women in their menstrual cycle, the collection was performed during cycle days 1 to 5. For serum separation, a centrifugation of the blood samples was applied for 10 min at 2.200x g. Systemic FSH was measured using solid-phase, enzyme-labeled chemiluminescent competitive immunoassay (IMMULITE 2000 XPi, Siemens Healthcare Diagnostics, UK).

A detailed description of the categorisation by menopausal status has been reported previously [20]. Briefly, participants’ menopausal status was characterised as *premenopausal* if they reported a regular menstrual cycle and had FSH values < 17 IU/L (M = 7.72, SD = 3.50); as *early perimenopausal* if they had FSH values from 17 to below 25 IU/L or if they reported an irregular menstrual cycle and had FSH values > 9.5 IU/L (M = 16.76, SD = 4.77); as *late perimenopausal* if they had FSH values in the range more or equal to 25 to 30 IU/L or if they reported occasional menstrual bleeding during the past 3 months and had FSH values > 30 IU/L (M = 44.90, SD = 19.96); and as *postmenopausal* if they reported no menstrual bleeding during the past 6 months and had FSH values > 30 IU/L or reported no menstrual bleeding during the past 3 months and had FSH values> 39 IU/L (M = 82.48, SD = 28.82).

*Menopausal symptoms* were assessed with a structured questionnaire to determine whether participants had any symptoms associated with menopause, such as: sweating, hot flashes, sleeping problems, headache, joint pain, tiredness, mood swings, vaginal symptoms, urinary track problems, sexual problems, or any other symptoms. For the present analysis, responses were recoded into a dichotomous variable (menopausal symptoms/no menopausal symptoms).

*Marital status* was assessed with the one question and responses were grouped as single, married or living with a partner, or divorced, separated or widowed. *Parity* was grouped as nulliparous, one or two children, and three or more children. *Employment status* was dichotomised as employed (paid or self-employed) or not regularly employed (study, unemployed, occasional job or retired). A diagnosed *mental disorder* was self-reported as yes/no. Information on the *use of medications* that could influence mental well-being (N05, psycholeptics and N06, psychoanaleptics) was self-reported and coded in accordance to the Anatomical Therapeutic Chemical Classification System [26]. The codes represent respective dichotomous variables – users or non-users.

### Data analysis

Characteristics of the participants are shown as means and standard deviations or as percentages. Multiple regression analyses were employed to examine the association between the physical performance variables and dimensions of mental well-being. In model 1, regression models were adjusted for body height, fat mass percentage, menopausal status and symptoms, marital status, parity, employment status, self-reported mental disorders, use of psycholeptics and psychoanaleptics. In model 2, physical activity was added. These variables, deemed theoretically important, were justified based on our previous study with ERMA participants on various associations between physical activity, physical performance measurements, and mental well-being [11, 12]. All analyses were performed with R, version 3.3.3 and the package sjmisc [27]. *P*-values ≤0.05 were considered statistically significant. All categorical variables included in the adjustment were treated automatically as categorical variables in the regression analyses.

## Results

The mean age of the participants was 51 (SD 2) years. More than half (56%) had secondary education and 42% had a tertiary degree (Table 1). Women were evenly distributed by their menopausal status. Most participants reported having experienced menopausal symptoms and were married or living in a registered partnership. More than half of the participants had one or two children and reported a medium level of physical activity. Nearly 90% were employed.

**Table 1:** Descriptive information on the participants

Variables	Total *n*	Mean (SD) or *n* (%)
Age, years	909	51.51 (2.05)
Education	898	
Primary		17 (2)
Secondary		506 (56)
Tertiary		375 (42)
Body height, m	908	1.62 (0.06)
Body mass, kg	908	69.73 (10.85)
Body fat mass, %	905	30.5 (7.48)
Menopausal status	909	
Premenopausal		232 (25)
Early perimenopausal		182 (21)
Late perimenopausal		197 (22)
Postmenopausal		298 (32)
Menopausal symptom (yes)	907	702 (77)
Marital status	906	
Single		79 (9)
Married or registered partnership		687 (76)
Divorced, separated or widowed		140 (15)
Parity	906	
Nulliparous		110 (12)
One or two		512 (55)
Three or more		284 (33)
Employment status	909	
Employed		808 (89)
Not regularly employed		101 (11)
Self-reported mental disorders (yes)	903	62 (7)
Users of psycholeptics (N05)	908	17 (2)
Users of psychoanaleptics (N06)	908	67 (7)
Physical activity level	909	
Low		159 (18)
Medium		570 (62)
High		180 (20)
Depressive symptoms, mean (SD)	908	0.46 (0.37)
Negative affect, mean (SD)	907	1.54 (0.50)
Positive affect, mean (SD)	908	3.78 (0.62)
Life satisfaction, mean (SD)	908	5.24 (1.07)
Hand grip strength, N	903	313.1 (59.6)
Knee extension strength, N	786	462.3 (94.6)
Vertical jumping height, m	813	0.19 (0.04)
Maximal walking speed (ms^−1^)	900	2.64 (0.46)
Six-minute walking distance (m)	835	669.1 (60.9)

Values given are n (%) for variables without units of measurement and mean (SD) for variables with units of measurement

### Depressive symptoms and negative affectivity

No significant associations between physical performance and depressive symptoms and negative affectivity were observed (Model 1, Tables 2 and 3). The results did not change after adding physical activity into the model (Model 2, Tables 2 and 3). However, physical activity showed a significant reverse association with depressive symptoms across all the physical performance predictors, indicating that, irrespective of physical performance, depressive symptoms were significantly lower in women with a self-reported medium or high physical activity level than in those with a low physical activity level. Physical activity was not significantly associated with negative affectivity.

**Table 2:** Associations of physical performance predictors with depressive symptoms

*Predictors*	Model 1			Model 2		
	*B*	*CI*	*p*	*B*	*CI*	*p*
Hand grip	0.03	−0.01 – 0.07	0.138	0.03	− 0.01 – 0.07	0.124
PA (medium level)^a^				−0.09	− 0.15 – − 0.03	**0.005**
PA (high level) ^a^				− 0.14	−0.22 – − 0.06	**0.001**
Knee extension	−0.01	− 0.03 – 0.02	0.536	0.00	−0.03 – 0.03	0.950
PA (medium level) ^a^				−0.09	− 0.16 – − 0.02	**0.011**
PA (high level) ^a^				−0.16	−0.24 – − 0.08	**0.001**
Vertical jumping height	−0.16	− 0.85 – 0.52	0.639	0.02	−0.68 – 0.71	0.960
PA (medium level) ^a^				−0.11	−0.18 – − 0.05	**0.001**
PA (high level) ^a^				−0.15	−0.24 – − 0.07	**< 0.001**
Maximal walking speed	0.03	−0.02 – 0.08	0.256	0.04	−0.02 – 0.09	0.168
PA (medium level) ^a^				−0.10	− 0.16 – − 0.03	**0.002**
PA (high level) ^a^				−0.14	−0.22 – − 0.07	**< 0.001**
Six-minute walking distance	0.01	−0.04 – 0.05	0.735	0.02	−0.03 – 0.07	0.395
PA (medium level)^a^				−0.08	− 0.14 – − 0.01	**0.021**
PA (high level) ^a^				−0.14	−0.22 – − 0.06	**0.001**

Model 1 adjusted for body height, fat mass %, menopausal status and symptoms, marital status, parity, employment status, self-reported mental disorders, use of psycholeptics, use of psychoanaleptics

Model 2 = Model 1+ physical activity

*B* unstandardised coefficients, *PA* physical activity

Values in bold indicate statistically significant results

^a^reference category is low physical activity level

**Table 3:** Associations of physical performance predictors with negative affectivity

*Predictors*	Model 1			Model 2		
	*B*	*CI*	*p*	*B*	*CI*	*p*
Hand grip	−0.00	− 0.06 – 0.05	0.902	− 0.00	− 0.06 – 0.05	0.903
PA (medium level)^a^				−0.01	−0.10 – 0.08	0.870
PA (high level) ^a^				0.01	−0.10 – 0.12	0.857
Knee extension	−0.03	−0.06 – 0.01	0.148	−0.03	− 0.07 – 0.01	0.133
PA (medium level)^a^				−0.02	−0.11 – 0.08	0.764
PA (high level) ^a^				0.00	−0.12 – 0.12	0.867
Vertical jumping height	−0.58	−1.55 – 0.40	0.245	−0.64	−1.64 – 0.35	0.207
PA (medium level)^a^				−0.01	− 0.10 – 0.09	0.898
PA (high level) ^a^				0.02	−0.09 – 0.14	0.693
Maximal walking speed	−0.01	−0.09 – 0.06	0.745	−0.01	− 0.09 – 0.06	0.733
PA (medium level)^a^				0.00	−0.09 – 0.09	0.980
PA (high level) ^a^				0.02	−0.09 – 0.13	0.765
Six-minute walking distance	−0.00	−0.07 – 0.06	0.920	−0.00	− 0.07 – 0.06	0.899
PA (medium level)^a^				−0.00	−0.10 – 0.09	0.966
PA (high level) ^a^				0.01	−0.11 – 0.13	0.876

Model 1 adjusted for height, fat mass %, menopausal status and symptoms, marital status, parity, employment status, self-reported mental disorders, use of psycholeptics, use of psychoanaleptics

Model 2 = Model 1+ Physical activity

*B* unstandardised coefficients, *PA* physical activity

Values in bold indicate statistically significant results

^a^reference category is low physical activity level

### Life satisfaction and positive affectivity

A significant positive association was observed between six-minute walking distance and life satisfaction (Model 1, Table 4). After adding physical activity into the model, this association remained significant (Model 2, Table 4). None of the other physical performance predictors were significantly associated with life satisfaction, although physical activity showed a significant positive association with life satisfaction for every physical performance predictor. This indicates that the physical performance of women with a medium or high physical activity level was also better than those with low physical activity.

**Table 4:** Associations of physical performance predictors with life satisfaction

*Predictors*	Model 1			Model 2		
	*B*	*CI*	*p*	*B*	*CI*	*p*
Hand grip	−0.03	− 0.14 – 0.09	0.664	− 0.03	−0.14 – 0.08	0.615
PA (medium level)^a^				0.36	0.18–0.54	**< 0.001**
PA (high level) ^a^				0.51	0.29–0.73	**< 0.001**
Knee extension	0.06	−0.02 – 0.13	0.149	0.03	−0.05 – 0.11	0.449
PA (medium level)^a^				0.37	0.18–0.57	**< 0.001**
PA (high level) ^a^				0.58	0.34–0.81	**< 0.001**
Vertical jumping height	0.57	−1.48 – 2.62	0.587	−0.12	−2.19 – 1.95	0.909
PA (medium level)^a^				0.40	0.21–0.59	**< 0.001**
PA (high level) ^a^				0.57	0.33–0.81	**< 0.001**
Maximal walking speed	0.10	−0.05 – 0.25	0.194	0.08	−0.07 – 0.23	0.312
PA (medium level)^a^				0.36	0.18–0.54	**< 0.001**
PA (high level) ^a^				0.52	0.29–0.74	**< 0.001**
Six-minute walking distance	0.19	0.05–0.32	**0.006**	0.15	0.01–0.28	**0.033**
PA (medium level)^a^				0.28	0.09–0.47	**0.004**
PA (high level) ^a^				0.47	0.23–0.71	**< 0.001**

Model 1 adjusted for height, fat mass %, menopausal status and symptoms, marital status, parity, employment status, self-reported mental disorders, use of psycholeptics, use of psychoanaleptics

Model 2 = Model 1+ Physical activity

*B* unstandardised coefficients, *PA* physical activity

Values in bold indicate statistically significant results

^a^reference category is low physical activity level

Significant positive associations were observed between knee extension, vertical jumping height, maximal walking speed, six-minute walking distance and positive affectivity (Model 1, Table 5). When physical activity was added into the model, these associations were no longer significant, except for that between six-minute walking distance and positive affectivity (Model 2, Table 5). Similarly, with respect to life satisfaction, physical activity showed a significant positive association with positive affectivity across all the physical performance predictors. These results indicate that, independently, physical activity is significantly positively associated with positive affectivity. However, of the investigated physical performance predictors, only six-minute walking distance had an independent positive association with positive affectivity.

**Table 5:** Associations of physical performance predictors with positive affectivity

*Predictors*	Model 1			Model 2		
	*B*	*CI*	*p*	*B*	*CI*	*p*
Hand grip	−0.01	−0.08 – 0.06	0.849	−0.01	−0.08 – 0.06	0.793
PA (medium level)^a^				0.19	0.08–0.29	**0.001**
PA (high level) ^a^				0.45	0.32–0.58	**< 0.001**
Knee extension	0.05	0.00–0.09	**0.041**	0.02	−0.02 – 0.07	0.366
PA (medium level)^a^				0.15	0.04–0.27	**0.007**
PA (high level) ^a^				0.41	0.28–0.55	**< 0.001**
Vertical jumping height	1.23	0.03–2.44	**0.045**	0.51	−0.69 – 1.71	0.403
PA (medium level)^a^				0.19	0.08–0.30	**0.001**
PA (high level) ^a^				0.44	0.30–0.58	**< 0.001**
Maximal walking speed	0.09	0.00–0.18	**0.046**	0.07	−0.02 – 0.16	0.105
PA (medium level)^a^				0.20	0.09–0.30	**< 0.001**
PA (high level) ^a^				0.46	0.33–0.60	**< 0.001**
Six-minute walking distance	0.16	0.08–0.24	**< 0.001**	0.12	0.04–0.20	**0.002**
PA (medium level)^a^				0.14	0.03–0.26	**0.011**
PA (high level) ^a^				0.41	0.28–0.55	**< 0.001**

Model 1 adjusted for height, fat mass %, menopausal status and symptoms, marital status, parity, employment status, self-reported mental disorders, use of psycholeptics, use of psychoanaleptics

Model 2 = Model 1+ Physical activity

*B*, unstandardised coefficients, *PA* physical activity

Values in bold indicate statistically significant results

^a^reference category is low physical activity level

## Discussion

The results showed that, among 47- to 55-year-old women, aerobic capacity (measured as six-minute walking distance) was, irrespective of physical activity, positively associated with life satisfaction and positive affectivity. Physical activity in turn showed a stronger association with positive mental well-being than that of physical performance assessed by muscle strength, muscle power and maximal walking speed. Physical performance was not associated with negative dimensions of mental well-being such as negative affectivity or depressive symptoms, whereas physical activity was associated with fewer depressive symptoms.

Although modest, the association of aerobic capacity with positive mental well-being was not attenuated when physical activity was included in the model. Although the association between physical activity and positive affect has been well demonstrated [28], studies have predominantly assessed short-term effects of physical activity on positive affect (e.g. after exercise interventions). In addition, physical performance level, which may play a role in affective responses to exercise [29], has often been overlooked in this relationship. Our study extends these results and provides evidence that in addition to regular leisure physical activity, aerobic capacity may have unique importance for positive mental well-being.

The fitness hypothesis holds that the beneficial effect of physical activity is due to gains in fitness [17]. It may be that, through regular physical activity, positive affective states accumulate over time [30] in parallel with improvements in aerobic physical fitness. Mechanistically, one biological pathway via which aerobic capacity may enhance positive affect is the greater oxidative capacity that results from improved mitochondrial function and angiogenesis, which in turn increases microvascular density in the brain [31]. This prompts speculation regarding structural and functional changes in the brain related specifically to mood state. However, due to the correlational study design, the opposite or bidirectional association is equally possible. Women with higher positive affect may be more physically active and hence fitter or their greater aerobic physical fitness may enable them to be more physically active.

Our results partially support those of the FLAMENCO study (the Fitness League Against MENopause Cost), which found a positive association between aerobic fitness and positive affect among perimenopausal women [32]. However, the same study also reported significant associations of self-reported muscle strength and speed-agility with positive and negative affect and depressive symptoms. This discrepancy could be explained by differences in the assessment methods. In comparison with performance-based measurements, self-reported physical performance may be less sensitive in identifying minor deficits in functioning because middle-aged participants may not yet recognize them.

Midlife represents a specific phase of life during which a reduction in physical activity can be expected [5, 19]. This may cause (or be a consequence of) vulnerability in mental well-being. Among middle-aged women, higher physical activity was associated with higher positive well-being and lower depressive symptomatology [12]. Thus, greater aerobic capacity may be viewed as a unique resource that helps to attenuate the effects of a decline in physical activity on mental well-being in middle-aged women.

Although aerobic capacity can be relatively quickly improved [33], it must be acknowledged that some individuals may have a genetic predisposition to high aerobic capacity [14]. In addition, genotype may also modify the association between physical activity and improvement in aerobic capacity [34]. This, however, raises the practical question of how to deal with individuals who have low aerobic capacity. Are there inherent reasons (such as an underlying medical condition) for low cardiorespiratory capacity or is it caused by modifiable factors (e.g., life-style choices) and correctable? Thus, our finding on the independent association of aerobic capacity and positive mental well-being, although promising for middle-aged women, merits further research (including other types of measurement of aerobic capacity) to confirm the direction of the association and identify the factors underlying it. Furthermore, it would be important to investigate, preferably using longitudinal data, competing models, where, for example, physical performance and physical activity were, alternatively, set as mediators.

In our study, other components of physical performance (low body muscle strength, muscle power, walking speed) were also associated with positive affectivity. However, these associations were attenuated after adjustment for physical activity. This result points to the role of physical activity in this association and may support the “mastery hypothesis”, according to which participation in physical activity may instil a sense of coherence and mastery resulting in increased positive affect [35, 36]. This sense of mastery may not necessarily require a high level of muscle strength or power, and it may also be a function of other socio-psychological factors of participation in physical activity [37].

Our study showed no associations between any of the physical performance measurements and either negative affect or depressive symptoms. Several systematic reviews, although not focusing on the concomitant role of physical activity, have shown that cardiorespiratory fitness associates with lower incidents of depression [38, 39]. We found that physical activity, not physical performance, was associated with fewer depressive symptoms. One recent study of women aged 45–69 years living in Singapore found that those with high depressive symptoms (CES-D ≥ 16) had lower handgrip strength and lower body muscle power (5 repeated chair stands) than those with no depressive symptoms [40]. Interestingly, the above-mentioned analysis was adjusted for physical activity, and hence may suggest that handgrip strength and lower extremity functioning are factors independent of a high level of depressive symptoms. We analysed the association between depressive symptoms and handgrip strength as continuous variables rather than the association high and low muscle strength groups. It is possible that the associations between the groups, which were formed by categorising the participants into two levels of depressive symptoms and two levels of handgrip strength, may have resulted in overestimation of the results.

The results indicate that the link between physical performance and depressive symptoms found in our participants may be explained by level of physical activity. If so, this suggests that the women in our study potentially derived social or psychological benefit from participation in physical activity even in the absence of a gain in muscle strength, power or walking speed. In support of this argument, previous studies have found that better physical health predicted physical activity in men but not in women, for whom the social context of physical activity may provide additional psychological benefit [41, 42]. However, the absence of sex differences in the performance vs. social benefits of physical activity has also been reported [43].

The main limitation of this study is the correlational analysis, which precluded us from drawing conclusions on causality. Another limitation is that physical activity was self-reported, and thus the number of highly physically active participants may have been overestimated [44]. Our study is limited to participants with BMI < 35 kg/m2. Obesity and menopausal factors may synergistically influence physical performance among obese middle-aged women [45]. Although, we controlled our analysis for menopausal status and fat mass percentage, our results may not be generalized to severely obese individuals.

Among the strengths of this study is the comprehensive measurement of physical performance in a large cohort study. Previous studies investigating the associations between physical performance and mental well-being outcomes have predominantly assessed aerobic fitness to the relative neglect of other components of physical performance. Moreover, they have largely explored the association between physical performance and depressive symptoms (i.e., the negative dimension of mental well-being). Our study extends previous research by including both positive as well as negative dimensions of mental well-being. We were also able to include confounders known to have associations with mental well-being or physical performance in middle-aged women, including menopausal status, which was carefully categorised using menstrual diaries and hormonal analysis following the STRAW+ 10 criteria, self-reported mental disorders and use of medications. Our study participants represent a homogeneous group of relatively healthy, non-severely obese women within a narrow age range, thereby reducing the possible influence of unobservable variables while also reducing the results to be generalised to more heterogeneous populations.

## Conclusions

This study, conducted among middle-aged women, revealed a positive association between aerobic capacity and positive mental well-being independently of the level of physical activity, although when physical activity was included in the analysis, the associations of the other components of physical performance studied (i.e., low body muscle strength, muscle power, walking speed) with positive affectivity were attenuated. Moreover, physical performance was not associated with the negative dimensions of mental well-being; instead, physical activity showed a stronger negative association with depressive symptoms. Thus, given the important role of physical activity for mental well-being found in this study, aerobic capacity may be considered a unique resource for positive mental well-being. However, the results warrant additional research to confirm the direction of the association and identify the factors underlying it.

## Data Availability

The dataset generated and analysed during the current study is not publicly available due to being confidential but is available from the PI of the project (Eija K. Laakkonen) on reasonable request.

## Abbreviations

PA: Physical Activity
FSH: Follicle Stimulating Hormone
ERMA: Estrogenic Regulation of Muscle Apoptosis
BMI: Body Mass Index
CES-D: Center for Epidemiological Studies Depression Scale
I-PANAS-SF: International Positive and Negative Affect Schedule Short Form
STRAW: Stages of Reproductive Aging Workshop
FLAMENCO: the Fitness League Against MENopause Cost
